# Food choice motives including sustainability during purchasing are associated with a healthy dietary pattern in French adults

**DOI:** 10.1186/s12937-017-0279-9

**Published:** 2017-09-18

**Authors:** B. Allès, S. Péneau, E. Kesse-Guyot, J. Baudry, S. Hercberg, C. Méjean

**Affiliations:** 1 Université Paris 13, Equipe de Recherche en Epidémiologie Nutritionnelle, Centre de Recherche en Epidémiologie et Statistiques, Inserm (U1153), Inra (U1125), Cnam, COMUE Sorbonne Paris Cité, F-93017 Bobigny, France; 2 Department of Public Health, Hôpital Avicenne, F-93300 Bobigny, France; 3 INRA, UMR 1110 MOISA, F-34000 Montpellier, France; 4 EREN (Nutritional Epidemiology Research Unit), SMBH Paris 13, 74 rue Marcel Cachin, F-93017 Bobigny Cedex, France

**Keywords:** Sustainability, Food choice motives, Diet, Dietary patterns

## Abstract

**Background:**

Sustainability has become a greater concern among consumers that may influence their dietary intake. Only a few studies investigated the relationship between sustainable food choice motives and diet and they focused on specific food groups.

**Objective:**

This cross-sectional study aimed to assess the associations between food choice motives during purchasing, with a focus on sustainability, and dietary patterns in a large sample of French adults.

**Design:**

Food choice motives were collected in 31,842 adults from the NutriNet-Santé study, using a validated 63 items questionnaire gathered into 9 dimension scores: ethics and environment, traditional and local production, taste, price, environmental limitation (i.e. not buying a food for environmental concerns), health, convenience, innovation and absence of contaminants. Dietary intake was assessed using at least three web-based 24-h food records. Three dietary patterns were obtained through factor analysis using principal component analysis. The associations between food choice motive dimension scores and dietary patterns were assessed using linear regression models, stratifying by sex.

**Results:**

Individuals were more likely to have a “healthy diet” when they were more concerned by not buying a food for environmental concerns (only for 3^rd^ tertile versus 1^st^ tertile β_women_=0.18, 95% CI=0.15–0.20, β_men_=0.20 95% CI=(0.15–0.25)), ethics and environment (women only, β=0.05, 95% CI=0.02–0.08), absence of contaminants (women only, β=0.05, 95% CI=0.01–0.07), local production (women only, β=0.08, 95% CI=0.04–0.11), health (women only) and innovation (men only), and when they were less concerned by price. Individuals were also less likely to have traditional or western diets when they gave importance to food choice motive dimensions related to sustainability.

**Conclusion:**

Individuals, especially women, having higher concerns about food sustainability dimensions such as ethics and environment and local production, appear to have a healthier diet. Further longitudinal studies are required to better understand how sustainable concerns may influence long-term nutritional quality of the diet.

**Electronic supplementary material:**

The online version of this article (10.1186/s12937-017-0279-9) contains supplementary material, which is available to authorized users.

## Background

Food sustainability has become a critical political issue in societies as well as a matter of public health. Sustainable diets were defined by the Food and Agriculture Organization (FAO) as “diets protective and respectful of biodiversity and ecosystems, culturally acceptable, accessible, economically fair and affordable; nutritionally adequate, safe and healthy; while optimizing natural and human resources” [1]. Considering that sustainable diets could be health promoting, scientific communities in USA [2], Brazil [3], UK [4] and some Northern countries from Europe [5], have recently proposed dietary guidelines taking sustainability into account. In fact, it has been previously reported that some current diets, such as a western diet rich in high fat, high sugar processed foods and poor in fruit and vegetables, has negative effects on both health and environment [6]. Consumers can be considered as the main stakeholders in nutritional public health policies [7]. Public health strategies aiming at encouraging healthy and environmentally friendly food choices need to better understand consumer motives when purchasing. However, little is known about the relationship between their food choice motives including those related to sustainability and dietary intake. Previous studies suggest that the motivation to behave sustainably is frequently found among consumers, while its translation into actual sustainable food choices and consumptions seems more difficult [8–13]. Previous researches about dietary behaviors have indicated that food choice motives may play a mediating role between personal norms and values and dietary behaviors [14, 15]. Indeed some types of concerns (e.g.: health, environment, etc.) may be explained by a combination of values that could influence dietary behaviors such as purchases and food choices and thus diet quality [15].

Previous studies about food choice motives conducted in industrialized countries identified price [16–19], health [17, 19, 20], sensory appeal [19, 21], mood during purchasing [19], attitude toward foods [22] convenience [16, 17, 19, 23–25] and ethical concerns [16, 17, 19, 23–25] as main motives influencing consumers choice. Although sustainability is a rising concern in consumers, to date, only a few studies [23, 25–29] have investigated food choice motives covering all the dimensions of sustainability as defined by the FAO [1]. Those studies investigated motives such as environmental, animal welfare, local production and they mainly focused on organic products [26–29] or specific food groups [23, 25]. For example, a study conducted in Finland reported that health and ethical concerns were associated with a higher consumption of fruit and vegetables and a lower consumption of energy dense foods [23]. Another study, conducted among young adults in the USA, reported that a positive attitude toward organically grown and local food was associated with a higher consumption of fruit and vegetables, and a lower consumption of sugar-sweetened beverages [29]. Finally, another study conducted in six European countries did not report any association between food choice motives related to sustainability and consumption of traditional foods, except for France [25].

To the best of our knowledge, the relationship between sustainable food choice motives and the overall diet such as dietary patterns has not been reported yet. Dietary patterns have the two advantages of reflecting the complexity of the dietary habits and overall food intake [30] and to enhance the promotion of healthy food habits [31]. In addition, specific dimensions of sustainable food choice motives such as health in the context of sustainability were rarely reported in previous studies [19, 25].

This cross-sectional study aimed to investigate the relationships between food choice motives including sustainability during purchasing, and dietary patterns in a large sample of French adults from the NutriNet-Santé Study.

## Material and methods

Subjects were participants in the NutriNet-Santé Study, a large web-based prospective observational cohort launched in France in May 2009 with a scheduled follow-up of 10 years. Participants were Internet-using adult volunteers from the general population aged 18 years or more. The study was designed to investigate determinants of dietary behaviours and nutritional status, as well as the relationships between nutrition and health. The design, methods and rationale of this study have been previously described [32]. Briefly, participants had to fill in an initial set of questionnaires assessing dietary intake, physical activity, anthropometry, lifestyle and socio-economic conditions along with health status to be included in the cohort. Each month, they are invited to fill out other questionnaires related to determinants of food behaviour and various nutritional and health status issues.

This study was conducted according to guidelines laid down in the Declaration of Helsinki, and all procedures were approved by the Institutional Review Board of the French Institute for Health and Medical Research (IRB Inserm n° 0000388FWA00005831) and the Commission Nationale Informatique et Libertés (CNIL n° 908,450 and n° 909,216). Written electronic informed consent to participate in the study was obtained from all subjects.

### Data collection

#### Assessment of food choice motives including sustainability

As no study has simultaneously and thoroughly explored all dimensions of sustainability as defined by FAO [1], in consumer food-buying motives, in particular social dimension, a new questionnaire including all aspects of sustainability has been developed [33].

In September 2013, 122,091 subjects living in the French metropolitan area were invited to complete this optional validated questionnaire on food choice motives on the “Nutrinet-Santé” website (https://www.etude-nutrinet-sante.fr/). The development and validation of the questionnaire have been previously described [33]. Briefly, the final questionnaire included the 63 most relevant items, and was divided into 9 dimensions scores obtained by first-order analysis: ethics and environment (17 items, e.g.: production waste, impact on earth’s resources, respect for working conditions), traditional and local production (12 items, e.g.: proximity of production, support for small-scale producers), taste (4 items), price (6 items), environmental limitations (4 items, e.g.: not buying meat for environmental reasons), health (6 items, e.g.: health impact, nutritional composition), convenience (4 items, e.g.: cooking convenience), innovation (4 items, e.g.: original of innovative product, innovative fabrication/conservation process) and absence of contaminants (5 items, e.g.: additives, exposure to chemicals). Four intercorrelated dimensions (ethics and environment, local and traditional production, health and absence of contaminants) formed a second-order factor interpreted as healthy and environmentally friendly consumption, corresponding to a sustainability dimension in food choices for consumers [33]. The other five uncorrelated dimensions do not directly relate to sustainable food choice motives.

Participants were first asked whether they were in charge of food supply in their household or not. Then, the questionnaire was divided into two main sections: one for general aspects of food purchasing and another one for purchasing of specific food groups (meat, fish, fruit and vegetable and dairy products). Participants were asked whether they were buying these products or not. If they were buying them, they answered all questions concerning their motives during purchasing. For each item, subjects were asked to rate on a 4-point Likert scale from “I strongly disagree” to “I strongly agree” (e.g.: “When I purchase [meat/fish/fruits and vegetables/dairy products], I take into account its impact on the environment: Strongly disagree / Disagree / Agree / Strongly agree / Undecided”). If they were not buying products from a food group, they had to answer specific questions on reasons for not buying them (e.g., “I avoid purchasing [meat/fish/fruits and vegetables/dairy products] for environmental issues: Strongly disagree / Disagree / Agree / Strongly agree / Undecided”).

To control the quality of the data collected through such a questionnaire, feasibility, internal validity and reliability were assessed in 637 randomly selected subjects participating in the Nutrinet-Santé cohort study [33]. Feasibility was measured by assessing specific questions on the perceived complexity and difficulty of filling in the questionnaire, and whether the questionnaire was too long and redundant, using the same 4-point Likert scale from « I strongly disagree » to « I strongly agree » [33]. The feasibility assessment revealed that only one third of the sample found the questionnaire redundant before it was shortened. The shorter version used for this study may be even more feasible.

The underlying structure of the questionnaire was determined by exploratory factor analysis and then internally validated by confirmatory factor analysis. Reliability was also assessed by internal consistency of selected dimensions and test–retest repeatability. The model demonstrated excellent internal validity (adjusted goodness of fit index = 0.97; standardized root mean square residuals = 0.07) and satisfactory reliability (internal consistency = 0.96, test–retest repeatability coefficient ranged between 0.31 and 0.68 over a mean 4-week period).

Also, to improve quality of data collected, controls were implemented in the web-based questionnaire to avoid missing values implying that individuals had to fill in every question. In addition, at the end of the questionnaire, participants had access to all questions and given answers to check if their answers were right and had the possibility to modify them eventually.

#### Dietary intake assessment

Dietary data were collected using web-based 24 h dietary records. At enrolment and yearly thereafter, participants were invited to provide three 24 h records (1 weekend day and 2 weekdays) [32]. These records were randomly assigned over a two-week period. The dietary record is completed via an interactive interface and designed for self-administration on the Internet [34]. The web-based dietary assessment method relies on a meal-based approach, recording all foods and beverages (type and quantity) consumed at breakfast, lunch, dinner and all other eating occasions. First, participants fills in the names of all food items eaten. Then, they estimate portion sizes for each reported food and beverage item according to standard measurements (e.g. home containers, grams displayed on the package) or using photographs available via the interactive interface. These photographs, taken from a validated picture booklet [35], represent more than 250 foods (corresponding to 1000 generic foods) served in seven different portion sizes. The values for energy were estimated using a published nutrient database [36] and completed for recent market foods and recipes. The accuracy of web-based 24 h dietary records has been assessed by comparing to interviews by trained dietitians [34] and against 24 h urinary biomarkers [37, 38].

Participants in our sample were included if they had completed at least three 24 h dietary records during the two years before the questionnaire on food choice motives (September 2011–September 2013). For each participant, daily mean quantities of the food group (in grams for solid food or cL for beverages) were calculated from 24 h records, weighted according to the day (week or weekend). Diet-underreporting participants were identified by the method proposed by Black [39]. Briefly, basal metabolic rate (BMR) was estimated by Schofield eqs. [40] according to sex, age, weight and height collected at enrolment in the study. BMR was compared to energy intake taking into account the physical activity level [39]. Foods were classified according to the information provided in the French Nutrition and Health Program (Programme National Nutrition Santé) guides [41]. Twenty-five food groups were created: fruit, vegetables, legumes, potatoes and other tubers, refined starchy foods, whole starchy foods, cereals (non fatty), fish and seafood, meat, eggs, processed meat, cheese, dairy products low in sugar, cream based deserts, butter and other added animal fats, vegetable oils, margarine, salad dressing and other dressings, salty snacks, cereals (sweet and fatty) sweet and fatty foods (pastries, biscuits, cookies, chocolate), sweet products (honey, jam, candy), sugary drinks, non-alcoholic beverages and alcoholic beverages.

### Statistical analysis

The present analyses focused on participants included in the NutriNet-Santé cohort study, included between May 2009 and October 2013, who completed the food choice motive questionnaires, who completed at least three 24 h dietary records, who are not diet-underreporting participants and with no missing socioeconomic data.

### Dietary patterns analysis

To create dietary patterns, factor analysis using principal component analysis was performed on the correlation matrix of the 25 food groups. Three principal components representing three independent dietary patterns were identified according to their eigenvalues, interpretability and percentage of variance explained. Varimax rotation was performed to improve the interpretability of the factor loadings [42]. Component scores of dietary patterns were then adjusted for energy using the residual method described by Willett and Stampfer [43]. Mean intakes of 25 food groups and products were described according to the quartiles of dietary pattern factor scores to allow better interpretation of the food intake associated with each one the dietary pattern obtained.

### Statistical analyses

Sociodemographic characteristics of the included and excluded samples were compared using chi^2^ tests and t-tests. Since each factor consisted of different number of items (from 17 to 4 items), all factors were linearly transformed into values ranging from 0 (no concern) to 10 (strong concern) to standardize ratings. Those quantitative score variables were later converted into qualitative variable to ease interpretation of the results. Cut-offs, were either tertiles or median, depending on the distribution of the score. The association between the nine food choice motives dimension scores and the three dietary pattern factor scores were assesssed using ANCOVA models. Each factor of dietary pattern identified was used as a dependent variable. Because of a statistcally significant interaction between sex and the food choices motives, the analyses were stratified by sex. First, an univariable analysis was performed for each dietary pattern, to select food choice motive dimensions that were significantly associated with dietary pattern (*p* < 0.05, data not shown). Then, a multivariable analysis assessed the associaton between dimensions of food motives and each dietary pattern. This analysis included the retained food choice motives dimensions in univariable models, adjusted for energy intake, age and education. Mean scores of the nine dimension scores of food choice motives were also computed by sex to describe their ranking in stratified analyses. Mean dimension scores were compared between men and women using t-tests.

All tests of significance were two-sided, and a *P* value <0.05 was considered significant. All statistical analyses were performed using SAS software (version 9.3, SAS Institute Inc.).

## Results

### Characteristics of the sample

From the initial 122,091 subjects who received the questionnaire measuring food choice motives regarding sustainable foods, a total of 46,958 answered the questionnaire. Then, 15,113 subjects were excluded because of missing data on food intake (including 6650 underreporting) and 3 had missing data for age and education. The final sample was composed by 31,842 subjects (25,217 women and 6625 men).

As shown in Table 1, in both sexes, the sample of included subjects had a higher proportion of individuals with higher education, compared with excluded subjects. The proportion of women living with a partner was higher in the included sample as well as those living in an urban area of 20,000 to 200,000 inhabitants. The mean age was also lower in included subjects than in excluded subjects.

**Table 1:** Characteristics of the included and excluded subjects (*n* = 45,155, Nutrinet-Santé study, 2013)

	Men (*n* = 9499)						Women (*n* = 35,656)					
	Excluded (*n* = 2874)			Included (*n* = 6625)		p^a^	Excluded (*n* = 10,439)			Included (*n* = 25,217)		p^a^
Age (mean SD)	54.4	13.3		51.4	13.9	<0.001	47.1	14.1		44.8	14.1	<0.001
Educational level (n %)						<0.001						<0.001
Primary	110	3.8		183	2.8		361	3.5		529	2.1	
Secondary	1070	37.2		2065	31.2		3756	36.0		7266	28.8	
Higher education	1685	58.6		4361	65.8		6263	60.0		17,302	68.6	
Missing data	9	0.3		16	0.2		59	0.6		120	0.5	
Marital status (*n* %)						0.78						0.001
Single, divorced, separated or widowed	578	20.1		1311	19.8		3002	28.8		6717	26.6	
Living with a partner	2289	79.6		5300	80.0		7420	71.1		18,456	73.2	
Missing data	7	0.2		14	0.2		17	0.2		44	0.2	
Size of the residential city (n %)						0.63						0.015
Rural	584	20.3		1395	21.1		2299	22.0		5519	21.9	
Paris area	542	18.9		1227	18.5		1952	18.7		4396	17.4	
Urban, 20,000–200,000 inhabitants	518	18.0		1253	18.9		1902	18.2		4660	18.5	
Urban, < 20,000 inhabitants	461	16.0		988	14.9		1551	14.9		3737	14.8	
Urban, > 200,000 inhabitants	762	26.5		1748	26.4		2694	25.8		6833	27.1	
Missing data	7	0.2		14	0.2		41	0.4		72	0.3	

^a^: *p*-value for t-test for age and for chi^2^ for every other categorical variables

*SD* Standard-deviation

### Description of food choice motive dimensions

Food choice motive dimensions ranked the same in men and women (Table 2). The highest mean scores were found for taste dimension, motives regarding health and absence of contaminants, followed by local and traditional production, price, ethics and environment and convenience. The lowest scores observed were for innovation and environmental limitations. Women had higher scores for every dimension except for innovation and environmental limitations.

**Table 2:** Mean food choice dimension scores in the sample, sorted from high to low (n = 31,842, Nutrinet-Santé study, 2013)

	Women		Men		p^1^
	Mean	SD	Mean	SD	
Taste	9.0	0.9	8.8	0.9	<0.001
Health	7.6	1.2	7.4	1.1	<0.001
Absence of contaminants	7.5	1.5	7.4	1.3	<0.001
Local and traditional production	7.4	1,0	7.2	0.8	<0.001
Price	7.4	1.1	7.1	1.1	<0.001
Ethics and environment	5.7	1,0	5.5	0.8	<0.001
Convenience	5.5	1.6	5.2	1.5	<0.001
Innovation	3.5	1.4	3.6	1.3	<0.001
Environmental limitations	2.8	2.2	2.7	2.2	0.22

1:*p*-value for t-test

s-d: standard-deviation

### Description of dietary patterns

Three dietary patterns explaining 24.9% of the total variance were derived (Table 3). The first dietary pattern was labelled healthy, it had the highest factor loadings for fruit, vegetables, legumes, whole starchy foods, fish and seafood, eggs, vegetable oils, non-alcoholic beverages and lowest factor loadings for cream based desserts, sweet and fatty food (pastries, biscuits, cookies and chocolate) and sugary drinks. The second dietary pattern was labelled traditional, it had the highest factor loadings for potatoes and other tubers, non-fatty cereals, meat, cheese, butter and other added animal fats, margarine and sweet products (honey, jam, candy) and the lowest factor loadings for fatty and sweet cereals. The third dietary pattern was labelled Western, it had the highest factor loadings for processed meat, cheese, salad dressings, salty snacks, sweet and fatty food, sugary drinks and alcoholic beverages and the lowest factor loadings for dairy products low in sugar and margarine. Mean daily intakes for food groups, according to quartiles of component scores obtained by FA-PCA, are presented in Additional file 1: Table S1.

**Table 3:** Dietary patterns obtained by factor analysis using principal component analysis of daily food intakes in the Nutrinet-Santé sample (*n* = 31,842, Nutrinet-Santé study, 2013)

	Healthy dietary pattern	Traditional dietary pattern	Western dietary pattern
Fruit	0.44	0.07	−0.21
Vegetables	0.58	0.20	−0.28
Legumes	0.27	−0.09	0.05
Potatoes and other tubers	0.04	0.42	0.00
Refined starchy food	−0.08	−0.06	0.18
Whole starchy food	0.60	−0.27	−0.01
Cereals, non fatty	−0.22	0.71	0.01
Fish and seafood	0.31	−0.07	0.02
Meat	−0.20	0.29	0.02
Eggs	0.14	0.05	−0.06
Processed meat	−0.11	0.24	0.40
Cheese	0.09	0.36	0.34
Dairy products, low in sugar	−0.13	0.05	−0.49
Cream based deserts	−0.20	−0.04	0.03
Butter and other added animal fats	0.08	0.43	−0.05
Vegetable oils	0.38	0.11	0.11
Margarine	0.04	0.24	−0.20
Salad dressings and other dressings	−0.02	0.10	0.28
Salty snacks	0.00	−0.04	0.48
Cereals, sweet and fatty	−0.01	−0.28	−0.07
Sweet and fatty foods (pastries, biscuits, cookies, chocolate)	−0.23	−0.07	0.37
Sweet products (honey, jam, candy)	0.13	0.38	0.02
Sugary drinks	−0.29	−0.10	0.30
Non-alcoholic beverages	0.44	0.01	0.04
Alcoholic beverages	0.12	0.22	0.53
variance explained (%)	8.8	8.2	7.9

### Associations between food choice motive dimension and dietary patterns

After univariable analyses, convenience dimension for the three dietary patterns and price dimension for the Western dietary pattern were excluded for multivariable analyses.

In both sexes, individuals with higher concern for environmental limitations (not buying specific food for environmental reasons), and those with lower concerns for price, were more likely to have a healthy dietary pattern (tertile 3 vs 1 only) (Tables 4 and 5). Women with higher concern for ethics and environment, those with higher interest in traditional and local production, health, and those with higher concern for absence of contaminants were more likely to have a healthy dietary pattern as well (Tables 4 and 5). Men with higher concern for innovation were also more likely to have a healthy dietary pattern. In both sexes, individuals with lower concerns for environmental limitations were more likely to have a traditional dietary pattern. In addition, women with moderate concern for health (tertile 2 vs 1 only), men with moderate concern for the absence of contaminants (tertile 2 vs 1 only), and men with lower concern for innovation, were more likely to have a traditional dietary pattern. Finally, in both sexes, individuals with lower concerns for health (in men, tertile 2 vs 1 only) were more likely to have a western dietary pattern. In addition, women with higher concern for taste (tertile 3 vs 1 only) and those with lower concern for health, and men with lower concern for environmental limitations, were more likely to have a western dietary pattern.

**Table 4:** Associations between dietary patterns and food choice motives dimension scores (*n* = 31,842, Nutrinet-Santé study, 2013)

	Healthy^a^				Traditional^a^			
	Women		Men		Women		Men	
	β	95% CI	β	95% CI	β	95% CI	β	95% CI
ethics and environment								
2nd tertile of score vs. 1st tertile	**0.047**	**[0.018; 0.076]**	−0.002	[−0.061; 0.058]	0.009	[−0.015: 0.034]	−0.007	[−0.065;0.050]
3rd tertile of score vs. 1st tertile	**0.033**	**[0.001; 0.065]**	−0.007	[−0.077; 0.064]	0.025	[−0.002: 0.052]	−0.037	[−0.106;0.030]
traditional and local production								
2nd tertile of score vs. 1st tertile	**0.080**	**[0.051; 0.109]**	0.013	[−0.046; 0.073]	0.008	[−0.016; 0.033]	0.007	[−0.050; 0.066]
3rd tertile of score vs. 1st tertile	**0.081**	**[0.049; 0.112]**	−0.019	[−0.089; 0.052]	−0.008	[−0.035; 0.018]	−0.009	[−0.078; 0.059]
taste								
2nd tertile of score vs. 1st tertile	0.016	[−0.010; 0.043]	0.041	[−0.018; 0.100]	−0.001	[−0.024; 0.020]	0.038	[−0.018; 0.096]
3rd tertile of score vs. 1st tertile	0.022	[−0.006; 0.050]	0.011	[−0.060; 0.083]	−0.011	[−0.035; 0.012]	−0.002	[−0.072; 0.067]
price								
2nd tertile of score vs. 1st tertile	−0.015	[−0.043; 0.013]	−0.025	[−0.086; 0.036]	−0.001	[−0.025; 0.020]	0.010	[−0.048; 0.069]
3rd tertile of score vs. 1st tertile	**−0.040**	**[−0.067; −0.013]**	**−0.074**	**[−0.141; −0.007]**	0.004	[−0.019; 0.027]	−0.014	[−0.079; 0.051]
health								
2nd tertile of score vs. 1st tertile	**0.042**	**[0.014; 0.071]**	0.003	[−0.059; 0.065]	**0.027**	**[0.002; 0.052]**	0.011	[−0.049; 0.072]
3rd tertile of score vs. 1st tertile	**0.069**	**[0.040; 0.099]**	**0.067**	**[0.001; 0.135]**	0.003	[−0.022; 0.029]	−0.059	[−0.125; 0.006]
absence of contaminants								
2nd tertile of score vs. 1st tertile	**0.046**	**[0.017; 0.074]**	0.017	[−0.044; 0.079]	−0.001	[−0.026; 0.023]	0.084	[0.024; 0.144]
3rd tertile of score vs. 1st tertile	**0.038**	**[0.008; 0.069]**	0.028	[−0.040; 0.095]	−0.019	[−0.045; 0.007]	−0.013	[−0.079; 0.052]
environmental limitations (above median vs. under median)	**0.175**	**[0.153; 0.198]**	**0.202**	**[0.149; 0.255]**	**−0.087**	**[−0.107; −0.068]**	**−0.130**	**[−0.181; −0.078]**
innovation (above median vs. under median)	−0.010	[−0.033;0.013]	**0.063**	**[0.010; 0.116]**	−0.011	[−0.031; 0.008]	**−0.061**	**[−0.113; −0.009]**

^a^: parameters estimated with multivariable linear regression models, 8 food choice dimension scores adjusted for age, education and total energy intake; in bold: statiscally significant

β: linear regression coefficient estimate; 95% CI = Confidence interval

**Table 5:** Associations between dietary patterns and food choice motives dimension scores (*n* = 31,842, Nutrinet-Santé study, 2013)

	Western^b^			
	Women		Men	
	β	95% CI	β	95% CI
ethics and environment				
2nd tertile of score vs. 1st tertile	0.014	[−0.013;0.041]	0.052	[−0.009; 0.114]
3rd tertile of score vs. 1st tertile	−0.014	[−0.043; 0.017]	0.001	[−0.071; 0.073]
traditional and local production				
2nd tertile of score vs. 1st tertile	−0.009	[−0.036; 0.017]	0.040	[−0.021; 0.103]
3rd tertile of score vs. 1st tertile	0.012	[−0.017; 0.042]	0.064	[−0.009; 0.014]
taste				
2nd tertile of score vs. 1st tertile	0.007	[−0.017; 0.033]	0.002	[−0.059; 0.063]
3rd tertile of score vs. 1st tertile	**0.037**	**[0.011; 0.064]**	0.046	[−0.028; 0.119]
health				
2nd tertile of score	**−0.051**	**[−0.078; −0.023]**	**−0.114**	**[−0.178; −0.049]**
3rd tertile of score	**−0.142**	**[−0.170; −0.114]**	−0.145	[−0.214; 0.075]
absence of contaminants				
2nd tertile of score	0.005	[−0.021; 0.033]	0.019	[−0.045; 0.083]
3rd tertile of score	−0.006	[−0.034; 0.023]	−0.001	[−0.070; 0.069]
environmental limitations (above median vs under median)	0.011	[−0.010; 0.032]	**−0.081**	**[−0.136; −0.026]**
innovation (above median vs under median)	−0.001	[−0.021; 0.033]	0.045	[−0.010; 0.099]

^b^: parameters estimated with multivariables linear regression models, 7 food choice dimensions scores + age, education and total energy intake; in bold: statiscally significant

β: linear regression coefficient estimate; 95% CI = Confidence interval; 95% CI = Confidence interval

## Discussion

To the best of our knowledge, this is the first study to investigate associations between sustainable food choice motives and dietary patterns. Individuals, and in particular women, having higher concerns about food sustainability motives such as ethics and environment, local and traditional production and health appear to have a healthier diet. Higher concern for taste was also associated with the traditional dietary pattern (in women) reflecting less healthy dietary habits.

Findings from previous studies support our results even if they focused on specific food groups instead of dietary patterns. They showed an association between sustainable food choice motives and fruit and vegetables [23, 29], lower consumption of high-fat high sugar food [29] and higher consumption of traditional food [25].

The positive association between sustainable food choice motives and a healthy diet may be explained by a combination of both egoistic and altruistic values that could influence food behaviour and diet [26, 44]. Indeed, egoistic motives, such as health, have been reported as better predictors of the purchase of foods [26] compared with altruistic motives.

In general, self-perception regarding health or ethics concerns can influence food motives that in turn will have an effect on dietary intake. A review on determinants of healthy eating reported that individuals defining themselves as health and environmental conscious, or animal friendly, for example, may have healthier dietary habits [45]. Another study showed that food choice motives play a mediating role in the relationship between health concerns of developing diseases, and healthy eating attitudes [14]. In particular, the latter study highlighted that food choice motives regarding natural content and ethical concerns may have a mediating role between health concern and healthy eating attitude.

In our study, food choice motives regarding ethics and environment and environmental limitations specifically, were positively associated with a healthy dietary pattern and negatively associated with a western dietary pattern. In addition, the environmental limitation dimension was negatively associated with the traditional dietary pattern. Our results are suggesting that individuals that have a healthy diet may be more concerned by the environmental impact of their diet, independently from health concerns. Individuals having concerns for environment may not have conflicting concerns with health. Indeed, in another sample from the Nutrinet-Santé study, we reported that having environmental concerns is not contradictory with adherence to nutritional guidelines (data not shown).

Personal norms, especially those regarding ethics in food production and protection of the environment may induce healthy eating [45], explaining the association with healthy dietary habits. Previous work from de Boer et al. [15] showed that ethical and environmental concerns may relate to personal values and value-related attitudes in a prevention motivational system, influencing food choice motives, and then, food intakes. According to the Higgin’s regulatory focus theory [46, 47], this motivational system is implying a higher level of concerns with safety and fulfilment of responsibilities, as well as concerns for security and avoidance of negative outcome. Following those theories, personal values such as ethics and environment protection may act as preventive psychological determinants of a healthier food behavior.

The traditional and local production dimension was also associated with a healthy dietary pattern. This dimension of food choice motives contains various concerns including proximity of production especially (for fruit and vegetables), artisanal or traditional production, support for small-scale producers (and cooperatives) or seasonality. Previous studies about food choice motives never assessed a dimension gathering those concerns.

Previous studies focusing on consumers' motives for buying local food highlighted that both organic and freshly locally grown characteristics of the products were important concerns [48, 49]. Indeed, individuals buying local food may have a particular interest in the seasonality and the origin of the products they buy, two personal values that may be on the motivational pathway to healthy eating attitudes [15]. This food choice motives dimension was not associated with the traditional dietary pattern. A possible explanation is that in our questionnaire, the traditional and local dimension was also representing altruistic values (e.g.: promoting local producers). These altruistic values may be associated with healthier diet [15] instead of traditional and less healthy dietary patterns.

Health dimension was positively associated with both healthy and traditional dietary patterns whereas it was inversely associated with a western dietary pattern. It has been shown that health as a food choice motive was positively correlated with better adherence to healthy nutritional guidelines [20]. The health concern, mediated by attitudes and personal values regarding the prevention of diseases or other health conditions, may be associated with healthier food choices as previously described [14].

The association between the absence of contaminants motive and a healthy dietary pattern observed in our study has not been previously assessed in the literature. This motive was also associated with a traditional dietary pattern in our study. A comparative study conducted across six European countries including France, investigated the relationship between food choice motives and consumption of traditional food [25]. In this study, natural content was a concern for consumers of traditional products [25]. Higher concerns for natural content [50] and natural products [14] were also previously related to higher consumptions of healthy food such as fruit and vegetables.

An association between the taste dimension and the western pattern in women was observed. Previous work that investigated food choice motives also reported that taste highly ranked among other dimensions of motives [19, 25, 27, 51]. Taste and food preferences (e.g.: fat, salt, sweet) have been reported as strong predictors of food intake [52–55]. The small variation of the taste dimension score within this sample may partially explain the absence of any other statistically significant association. This may be also be explained by the conceptual model proposed by De Boer et al. [15] implying that food choice motives influence taste-related attitudes and not directly food choices.

Lower concern for price, independently from other dimensions, is associated with healthy food intake. Previous study reported that food price is an obvious determinant of food choices [19] and a food choice motive positively associated with healthy eating [14]. However, to the best of our knowledge, lower concerns for price were never reported associated with healthy dietary patterns in previous studies. Individuals with healthier diet have higher incomes in the Nutrinet-Santé study (data not shown) and, they may therefore feel less concerned by price. This may explain the inverse relationships between price motive and healthy dietary pattern.

The innovation dimension was poorly associated with any of the dietary patterns, except with a healthy dietary pattern in men. The fact that individuals having those concerns have higher incomes, higher educational level and thus, healthier diets (data not shown), may explain this significant association with a healthy dietary pattern in men, similarly to price concerns.

Our results may not be generalizable to the general population. First, our study sample includes a greater proportion of women and participants with higher education. Those characteristics that have been previously reported as demographic predictors of greater concerns for health and sustainability [29, 56, 57]. Indeed, a previous study among older adults reported that individuals with higher income and higher level of educations were more likely to report health related motives [56]. Another studies highlighted that women were more likely to exhibit higher concerns for the environment, whereas individuals with lower levels of educations are less environmentally sensitive [57]. Women seemed to also report higher importance for local production and sustainable foods in a previous study among young adults [29]. Thus, individuals with greater concerns for health and sustainability may be over-represented in this sample. However, it may be difficult to estimate how it could have biased our study sample, as no data from studies using random samples in the general population are available. To prevent this bias, statistical models were stratified by sex and adjusted for education.

Secondly, a previous study assessed the representativity of the Nutrinet-Santé cohort study comparing the distribution of sociodemographic and economic characteristics to statistics from the French census data (58). Notable differences were reported concerning gender and educational level. Thus, women and individuals with higher level of education are over-represented in our sample from the Nutrinet-Santé study, as they are more likely to participate in voluntary-based health and epidemiological studies in many epidemiological fields [58]. A high interest in nutrition could also lead to this overrepresentation [58, 59].

Finally, a comparison study about dietary intake was conducted comparing the Nutrinet-Santé study cohort to a representative sample of the French population [60]. The authors reported a low magnitude of differences in food intakes between those studies, except for fruit and vegetables.

Food consumptions were self-reported using a self-administrated web-based tool, implying methodological constraints. However, a validation study [61] concluded that compared to interview with dietitian, web-based self-reported food intake seem to be valid and feasible method. A strength of this study is to provide an accurate estimation of overall dietary intakes. Indeed, dietary data used to derive dietary patterns were collected through a validated [37] interactive web-based self-reported dietary record tool. Moreover, the three non-consecutive-day dietary records are recommended methods in large epidemiological studies [62], especially because it allows a good estimation of usual diet [63]. Additionally, we used a data-driven method that enables the description of global dietary patterns instead of focusing on specific food items. This method enhanced a broader description of food habits within the sample that were later associated with food choice motives. Within our study sample, we were able to cover a good variety of food habits, and related dietary patterns, similar to previous studies using these methods (for example: opposite nutritional qualities of healthy and western diets [64, 65]).

Food choice motives were assessed using a validated questionnaire specifically designed for the French population [33]. As food choice motives were self-reported some difficulties may have appeared when participants completed it by themselves [33]. As the answers from the questionnaire were based on self-reporting, the reliability and validity of the questionnaire could be questioned. However, reliability tests were performed [33] and showed that both internal consistency for each factor and repeatability for most of the items were satisfying. In addition, the model demonstrated excellent internal validity. However, external validity may be limited because this questionnaire was developed in a French cultural setting and cross-cultural adaptations may be required before submitting it to other cultures [24]. To our knowledge, this is the first study using such a validated food choice motives questionnaire with a specific focus on sustainability. Indeed, additionally to an increasing number of items investigated compared to previous studies, this questionnaire also covered new themes such as local production and environmental limitations for example.

## Conclusion

In some countries, public health experts are already promoting sustainable diets that could allow reaching a better nutritional quality of the diet, but little is known about determinants of consumers’ choices concerning sustainability. Our results support the idea that sustainable food motives during puchases are related to healthier dietary patterns. We also highlighted that sustainable concerns may influence dietary intake of individuals, and thus should not be neglected in the promotion of healthy dietary habits. Further longitudinal observational studies are also required to better understand how sustainable concerns may influence long term nutritional quality of diets.

## Additional file

Additional file 1: Table S1. Mean daily intakes for food groups according to quartiles of component scores obtained by factor analysis - principal component analysis of food intake data in the Nutrinet-Santé sample (*n* = 31,842, Nutrinet-Santé study, 2013). (DOCX 55 kb)

## Abbreviations

BMR: Basal metabolic rate
FAO: Food and Agriculture Organization
FA-PCA: Factor analysis using principal component analysis
