# Supplementary material for: Food choice motives including sustainability during purchasing are associated with a healthy dietary pattern in French adults
Source: Nutr J. 2017 Sep 18;16:58. doi: 10.1186/s12937-017-0279-9 (PMC5604508; doi:10.1186/s12937-017-0279-9)
Supplement: Additional file 1: Table S1. — Mean daily intakes for food groups according to quartiles of component scores obtained by factor analysis - principal component analysis of food intake data in the Nutrinet-Santé sample (n = 31,842, Nutrinet-Santé study, 2013). (DOCX 55 kb) [file 12937_2017_279_MOESM1_ESM.docx]

Additional file 1 Table S1. Mean daily intakes for food groups according to quartiles of component scores obtained by factor analysis - principal component analysis of food intake data in the Nutrinet-Santé sample (n=31,842, Nutrinet-Santé study, 2013)

| Food groups^1^ | Healthy | | | | |  | Traditionnal diet | | | | |  | Western | | | | |
| --- | --- | --- | --- | --- | --- | --- | --- | --- | --- | --- | --- | --- | --- | --- | --- | --- | --- |
|  | Q1 | Q2 | Q3 | Q4 | p^2^ |  | Q1 | Q2 | Q3 | Q4 | p^2^ |  | Q1 | Q2 | Q3 | Q4 | p^2^ |
| Fruit | 179.5 | 240.4 | 284.3 | 361.8 | <0.001 |  | 278.3 | 260.8 | 259.4 | 267.6 | <0.001 |  | 334.9 | 282.4 | 247.0 | 201.6 | <0.001 |
| Vegetables | 169.0 | 231.1 | 281.0 | 376.2 | <0.001 |  | 239.5 | 247.4 | 265.7 | 304.8 | <0.001 |  | 336.8 | 278.3 | 239.7 | 202.7 | <0.001 |
| Legumes | 6.2 | 9.3 | 12.2 | 22.4 | 0.003 |  | 18.3 | 12.2 | 10.1 | 9.4 | <0.001 |  | 11.1 | 12.6 | 13.2 | 13.2 | <0.001 |
| Potatoes and other tubers | 46.6 | 46.4 | 46.9 | 48.2 | <0.001 |  | 28.3 | 37.3 | 47.4 | 75.0 | <0.001 |  | 54.4 | 47.6 | 43.5 | 42.5 | <0.001 |
| Refined starchy food | 73.5 | 64.7 | 59.9 | 56.0 | <0.001 |  | 79.5 | 64.5 | 58.6 | 51.5 | <0.001 |  | 55.0 | 60.0 | 65.3 | 73.8 | <0.001 |
| Whole starchy food | 10.5 | 20.5 | 36.6 | 80.3 | <0.001 |  | 63.4 | 40.3 | 27.9 | 16.4 | <0.001 |  | 40.5 | 38.7 | 36.4 | 32.4 | <0.001 |
| Cereals, non fatty | 97.9 | 89.8 | 76.2 | 52.2 | <0.001 |  | 39.2 | 57.8 | 83.8 | 135.3 | <0.001 |  | 94.3 | 78.2 | 72.9 | 70.7 | <0.001 |
| Fish and seafood | 97.3 | 84.6 | 77.1 | 64.7 | <0.001 |  | 64.8 | 77.1 | 85.4 | 96.5 | <0.001 |  | 88.2 | 80.0 | 76.5 | 79.0 | <0.001 |
| Meat | 10.3 | 12.8 | 15.1 | 17.3 | <0.001 |  | 13.2 | 13.8 | 14.5 | 14.1 | <0.001 |  | 16.9 | 14.5 | 12.7 | 11.5 | <0.001 |
| Eggs | 26.3 | 37.6 | 48.8 | 62.1 | <0.001 |  | 50.7 | 45.0 | 42.1 | 37.1 | <0.001 |  | 44.5 | 43.3 | 43.1 | 44.0 | 0.54 |
| Processed meat | 23.7 | 21.0 | 18.1 | 14.1 | <0.001 |  | 15.3 | 17.8 | 19.9 | 24.0 | <0.001 |  | 10.8 | 14.4 | 19.8 | 32.0 | <0.001 |
| Cheese | 34.9 | 37.5 | 38.4 | 38.0 | <0.001 |  | 30.6 | 34.2 | 38.2 | 45.9 | <0.001 |  | 29.6 | 34.2 | 38.5 | 46.5 | <0.001 |
| Dairy products, low in sugar | 209.9 | 170.0 | 159.8 | 147.1 | <0.001 |  | 174.6 | 170.7 | 168.9 | 172.6 | 0.33 |  | 314.7 | 172.5 | 118.2 | 81.4 | <0.001 |
| Cream based deserts | 51.5 | 34.4 | 26.7 | 21.3 | <0.001 |  | 41.9 | 35.7 | 30.7 | 25.7 | <0.001 |  | 33.1 | 35.5 | 34.0 | 31.3 | 0.007 |
| Butter and other added animal fats | 13.8 | 14.9 | 15.2 | 15.8 | <0.001 |  | 9.5 | 12.5 | 15.5 | 22.2 | <0.001 |  | 17.7 | 15.9 | 13.8 | 12.2 | <0.001 |
| Vegetable oils | 5.8 | 7.3 | 9.2 | 13.4 | <0.001 |  | 9.2 | 8.6 | 8.6 | 9.3 | 0.71 |  | 8.5 | 8.9 | 9.0 | 9.3 | <0.001 |
| Margarine | 2.0 | 2.1 | 2.4 | 2.4 | <0.001 |  | 1.1 | 1.6 | 2.2 | 4.2 | <0.001 |  | 4.5 | 2.1 | 1.4 | 1.0 | <0.001 |
| Salad dressings and other dressings | 18.8 | 18.3 | 17.9 | 16.4 | <0.001 |  | 18.1 | 17.3 | 17.9 | 18.1 | 0.83 |  | 13.4 | 15.8 | 18.2 | 24.0 | <0.001 |
| Salty snacks | 4.2 | 4.1 | 3.9 | 3.6 | <0.001 |  | 5.8 | 4.1 | 3.3 | 2.6 | <0.001 |  | 0.8 | 1.7 | 3.0 | 10.4 | <0.001 |
| Cereals, sweet and fatty | 6.9 | 5.5 | 5.4 | 6.0 | <0.001 |  | 15.1 | 5.2 | 2.4 | 1.2 | <0.001 |  | 8.5 | 6.7 | 5.1 | 3.7 | <0.001 |
| Sweet and fatty foods (pastries, biscuits, cookies, chocolate) | 96.2 | 72.1 | 59.4 | 47.9 | <0.001 |  | 98.1 | 72.5 | 58.6 | 46.5 | <0.001 |  | 51.1 | 62.6 | 74.6 | 87.3 | <0.001 |
| Sweet products (honey, jam, candy) | 20.7 | 23.2 | 24.9 | 26.0 | <0.001 |  | 16.9 | 19.9 | 24.6 | 33.5 | <0.001 |  | 26.2 | 24.8 | 23.0 | 20.8 | <0.001 |
| Sugary drinks | 85.3 | 33.0 | 22.9 | 15.6 | <0.001 |  | 68.5 | 38.3 | 28.8 | 21.3 | <0.001 |  | 17.3 | 25.4 | 38.6 | 75.6 | <0.001 |
| Non-alcoholic beverages | 87.0 | 108.0 | 123.2 | 150.0 | <0.001 |  | 119.7 | 117.3 | 116.6.5 | 114.6 | <0.001 |  | 115.7 | 117.4 | 117.9 | 117.1 | 0.14 |
| Alcoholic beverages | 7.6 | 9.9 | 11.2 | 11.5 | <0.001 |  | 72.3 | 86.7 | 99.3 | 13.9 | <0.001 |  | 3.8 | 5.8 | 9.1 | 21.1 | <0.001 |

1: solid food in g per day and beverages in cL per day 2: p for linear trend Q: Quartile
